# The Influence of Educational Experiences and Role Models on Junior Doctors’ Decision to Specialise in Psychiatry

**DOI:** 10.1192/bjo.2026.11293

**Published:** 2026-06-30

**Authors:** Laura Pearson, Rebecca Williams

**Affiliations:** 1 Cardiff University, Cardiff, United Kingdom; 2 Swansea Bay University Health Board, Swansea, United Kingdom

## Abstract

**Aims:**

There have been long-standing challenges associated with recruitment into psychiatry internationally. In the UK, this issue has inevitably led to a shortage of practising mental health consultants which impacts upon training opportunities, patient care and staff morale. This study aims to explore existing psychiatric trainees’ perspectives on their career choice and the educational experiences that impacted upon their decision.

**Methods:**

This is a qualitative research study using semi-structured interviews to explore the perceptions of trainees within psychiatry on their educational journey and the interactions with role models that ultimately influenced their career aspirations. Thematic analysis was used to organise the findings into three overarching themes and various subthemes that aimed to interpret and explain meaning across the dataset.

**Results:**

Thirteen participants were interviewed demonstrating a range of ideas that were broadly grouped into three main themes. Theme one, entitled attitudes and behaviours towards and within psychiatry encompassed the importance of role models within psychiatry, a humanistic approach to patient care and to trainees themselves, and the trainees' experiences of professional stigma. Theme two, influential educational experiences within psychiatry explored the impact of specific teaching and learning experiences, potential barriers and unique and influential opportunities available. Theme three, perspectives and perceptions of psychiatry as a specialty concerned the trainees’ perceptions of how others viewed them as psychiatrists and involved discussion of common misconceptions, access to the specialty prior to application and visibility of the specialty in other contexts.

**Conclusion:**

Exploring trainees’ experiences in the lead up to choosing psychiatry as a career provided valuable insight into existing positive experiences and highlighted aspects of learning and training that could be further developed. The results can be used to provide a platform from which to enhance the educational experiences of future potential psychiatrists.

